# Supplementary material for: A Humanized Yeast Phenomic Model of Deoxycytidine Kinase to Predict Genetic Buffering of Nucleoside Analog Cytotoxicity
Source: Genes (Basel). 2019 Sep 30;10(10):770. doi: 10.3390/genes10100770 (PMC6826991; doi:10.3390/genes10100770)
Supplement: Supplementary file 1 [file genes-10-00770-s001.zip › Additional_Files/Additional_File7_GOTermHeatmaps.zip!/Additional_File7_GOTermHeatmaps/regulation_of_vesicle-mediated_transport.pdf]

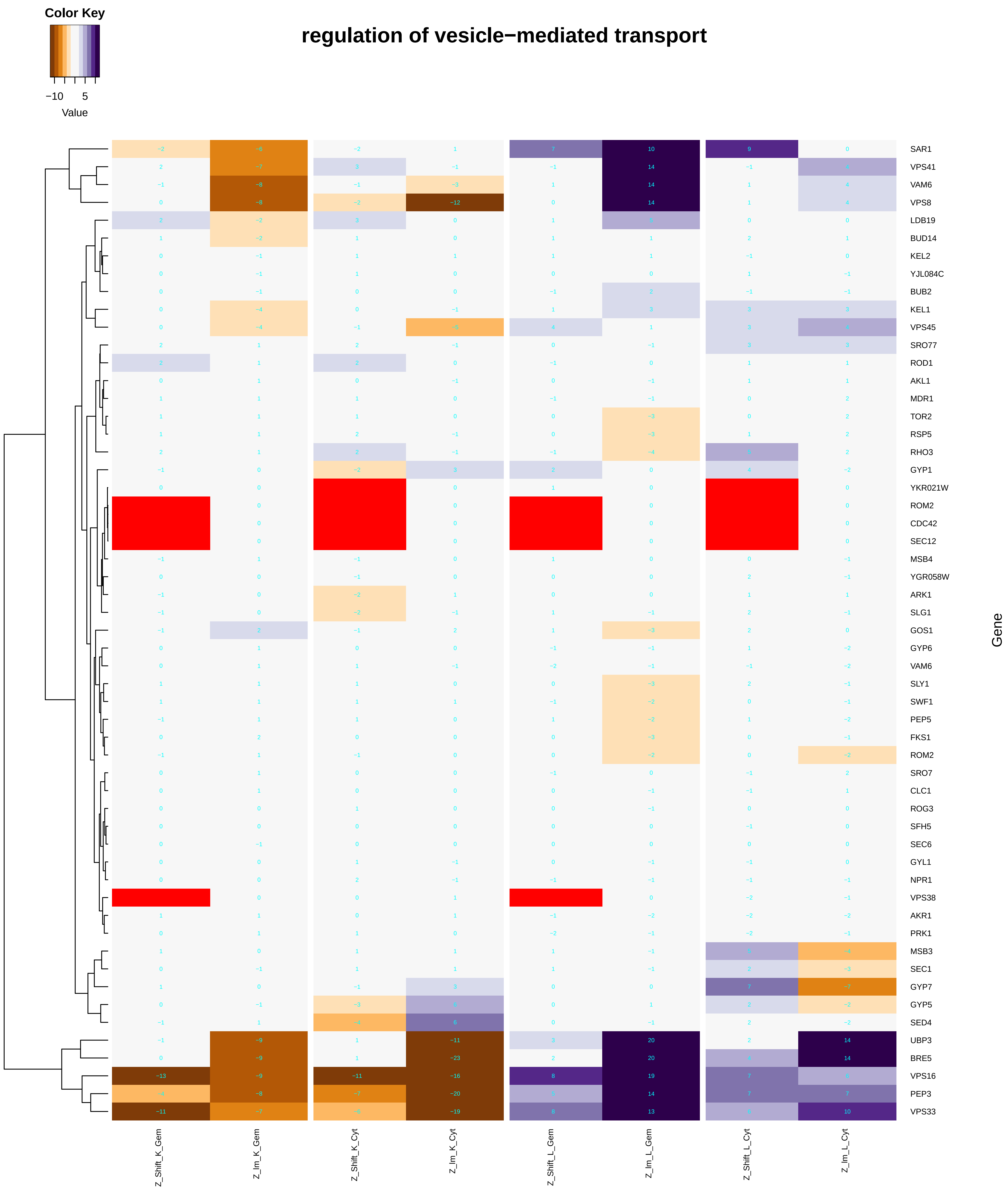

regulation of vesicle−mediated transport
Color Key
−10
5
Value
Gene
SAR1
VPS41
VAM6
VPS8
LDB19
BUD14
KEL2
YJL084C
BUB2
KEL1
VPS45
SRO77
ROD1
AKL1
MDR1
TOR2
RSP5
RHO3
GYP1
YKR021W
ROM2
CDC42
SEC12
MSB4
YGR058W
ARK1
SLG1
GOS1
GYP6
VAM6
SLY1
SWF1
PEP5
FKS1
ROM2
SRO7
CLC1
ROG3
SFH5
SEC6
GYL1
NPR1
VPS38
AKR1
PRK1
MSB3
SEC1
GYP7
GYP5
SED4
UBP3
BRE5
VPS16
PEP3
VPS33
Z_Shift_K_Gem
Z_Im_K_Gem
Z_Shift_K_Cyt
Z_Im_K_Cyt
Z_Shift_L_Gem
Z_Im_L_Gem
Z_Shift_L_Cyt
Z_Im_L_Cyt

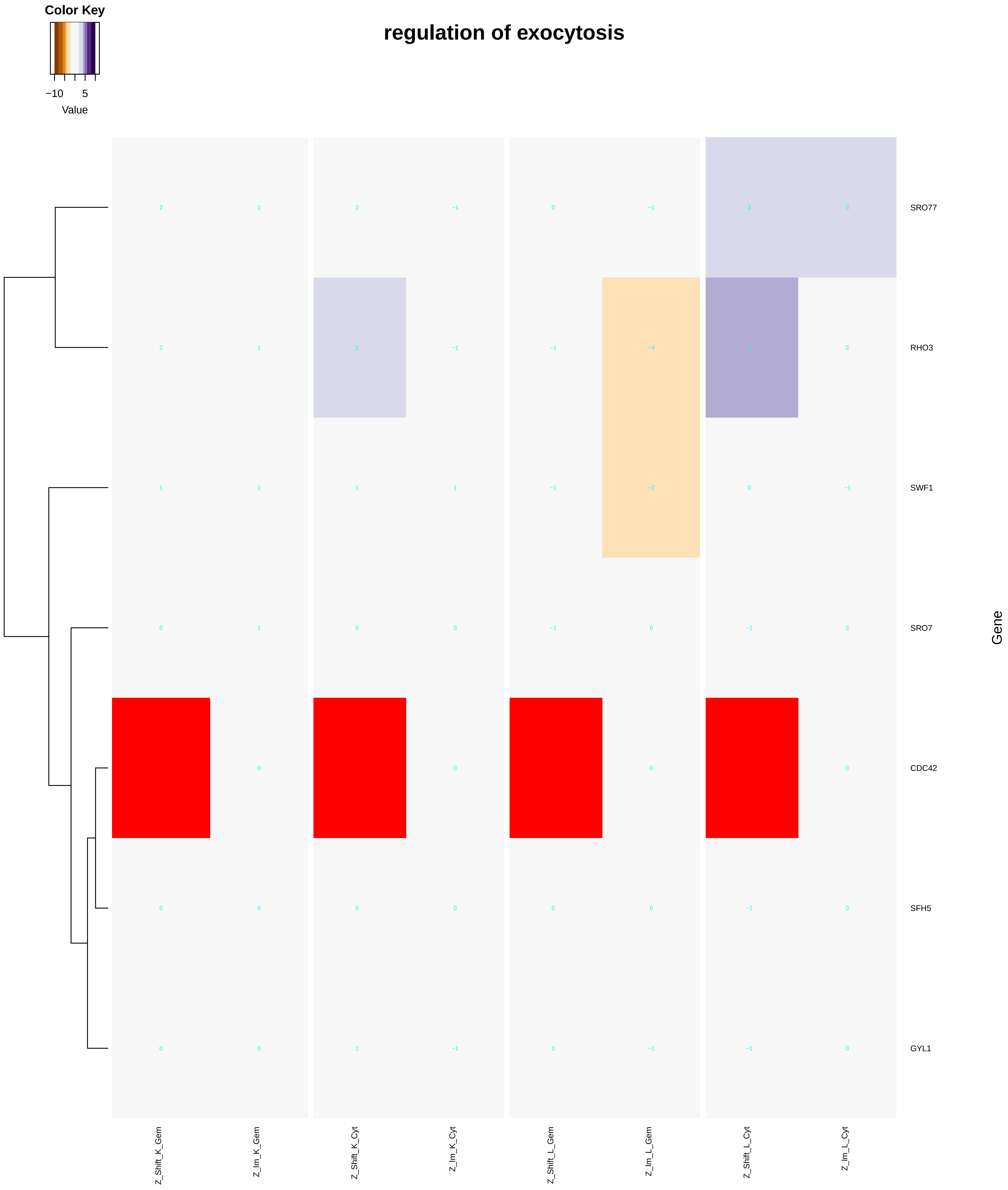

Color Key
−10
5
Value
regulation of exocytosis
SRO77
RHO3
SWF1
SRO7
CDC42
SFH5
GYL1
Gene
Z_Shift_K_Gem
Z_Im_K_Gem
Z_Shift_K_Cyt
Z_Im_K_Cyt
Z_Shift_L_Gem
Z_Im_L_Gem
Z_Shift_L_Cyt
Z_Im_L_Cyt

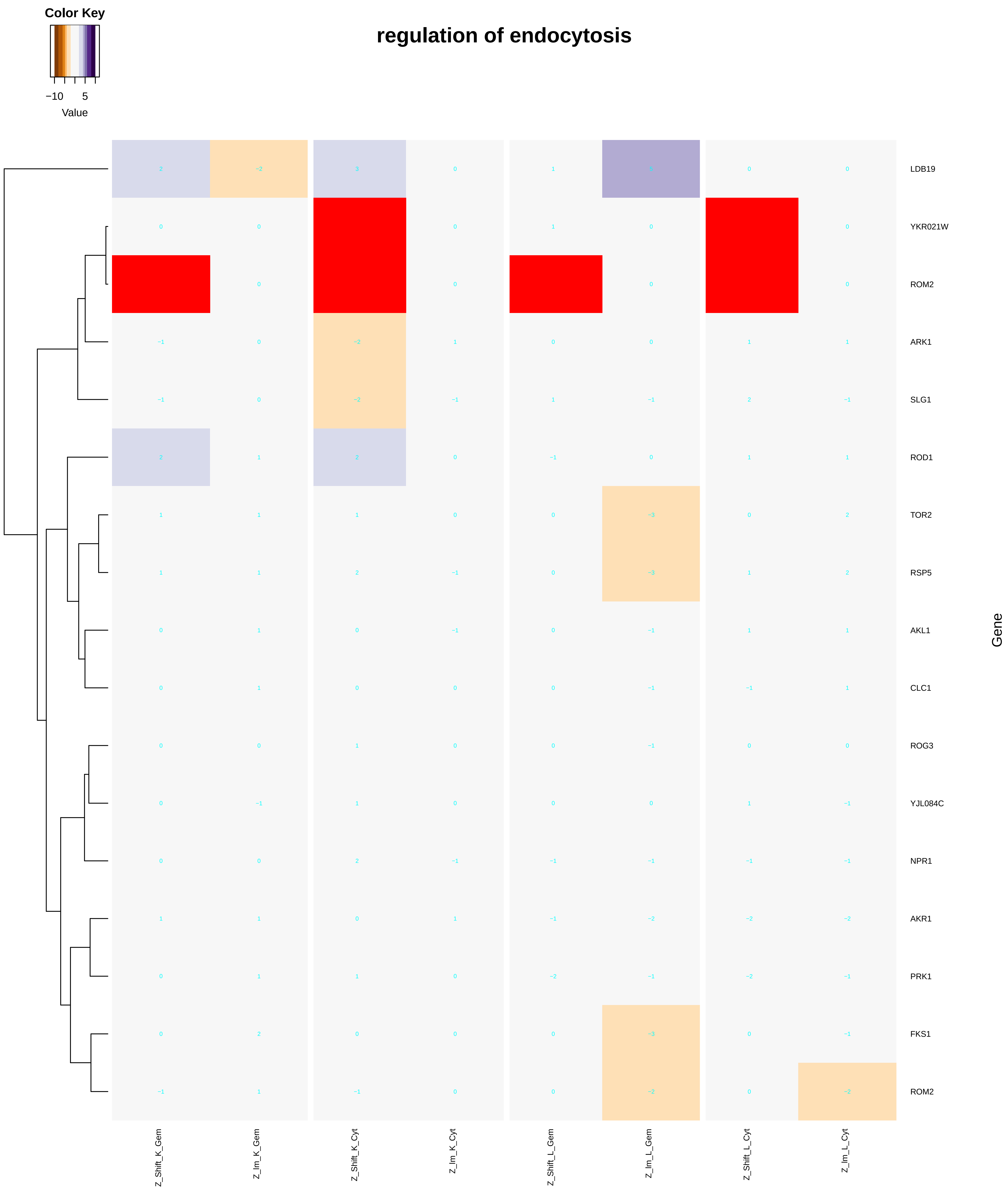
Color Key
−10
5
Value
regulation of endocytosis
LDB19
YKR021W
ROM2
ARK1
SLG1
ROD1
TOR2
RSP5
AKL1
CLC1
ROG3
YJL084C
NPR1
AKR1
PRK1
FKS1
ROM2
Gene
Z_Shift_K_Gem
Z_Im_K_Gem
Z_Shift_K_Cyt
Z_Im_K_Cyt
Z_Shift_L_Gem
Z_Im_L_Gem
Z_Shift_L_Cyt
Z_Im_L_Cyt

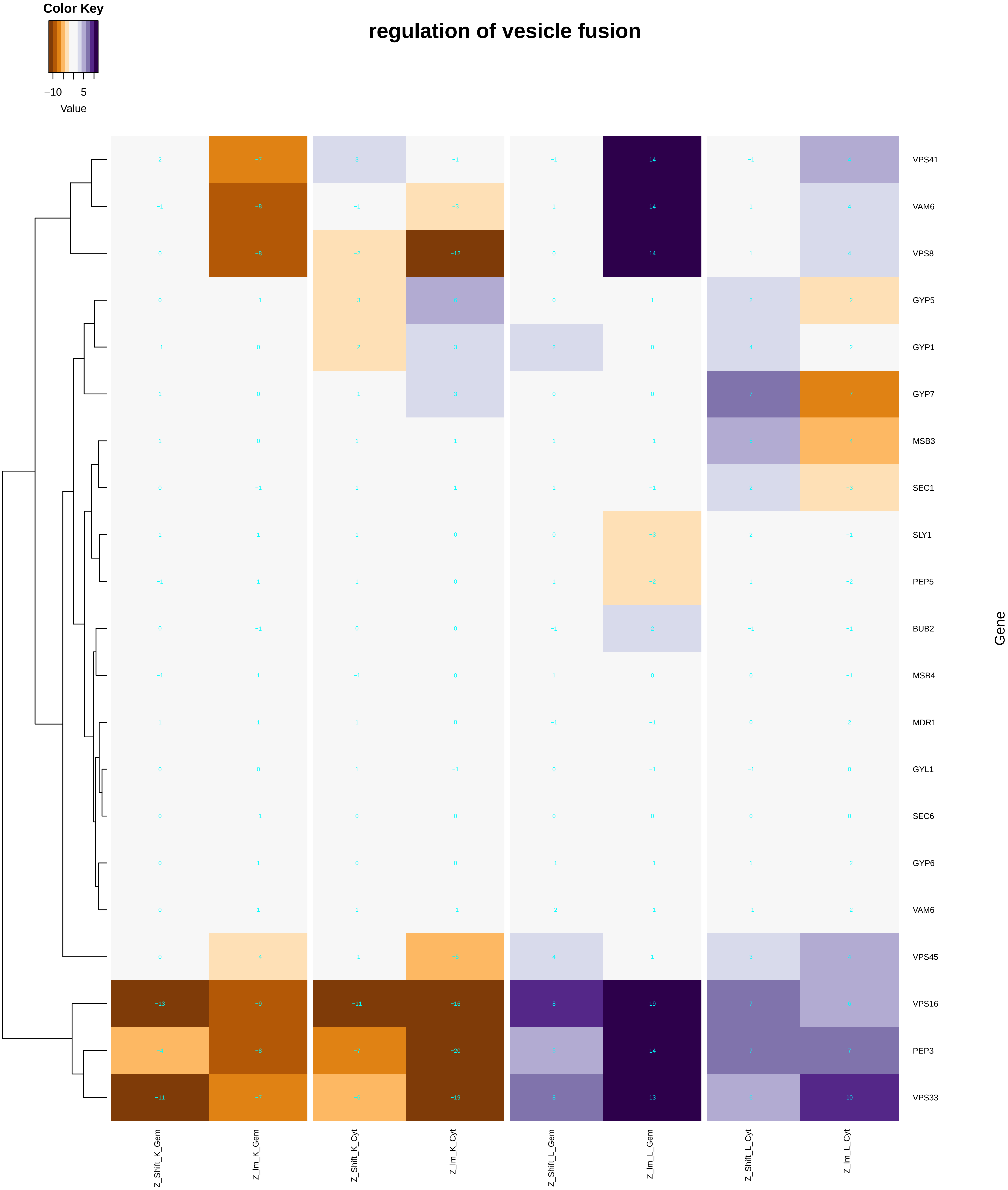

Color Key
−10
5
Value
regulation of vesicle fusion
VPS41
VAM6
VPS8
GYP5
GYP1
GYP7
MSB3
SEC1
SLY1
PEP5
BUB2
MSB4
MDR1
GYL1
SEC6
GYP6
VAM6
VPS45
VPS16
PEP3
VPS33
Gene
Z_Shift_K_Gem
Z_Im_K_Gem
Z_Shift_K_Cyt
Z_Im_K_Cyt
Z_Shift_L_Gem
Z_Im_L_Gem
Z_Shift_L_Cyt
Z_Im_L_Cyt

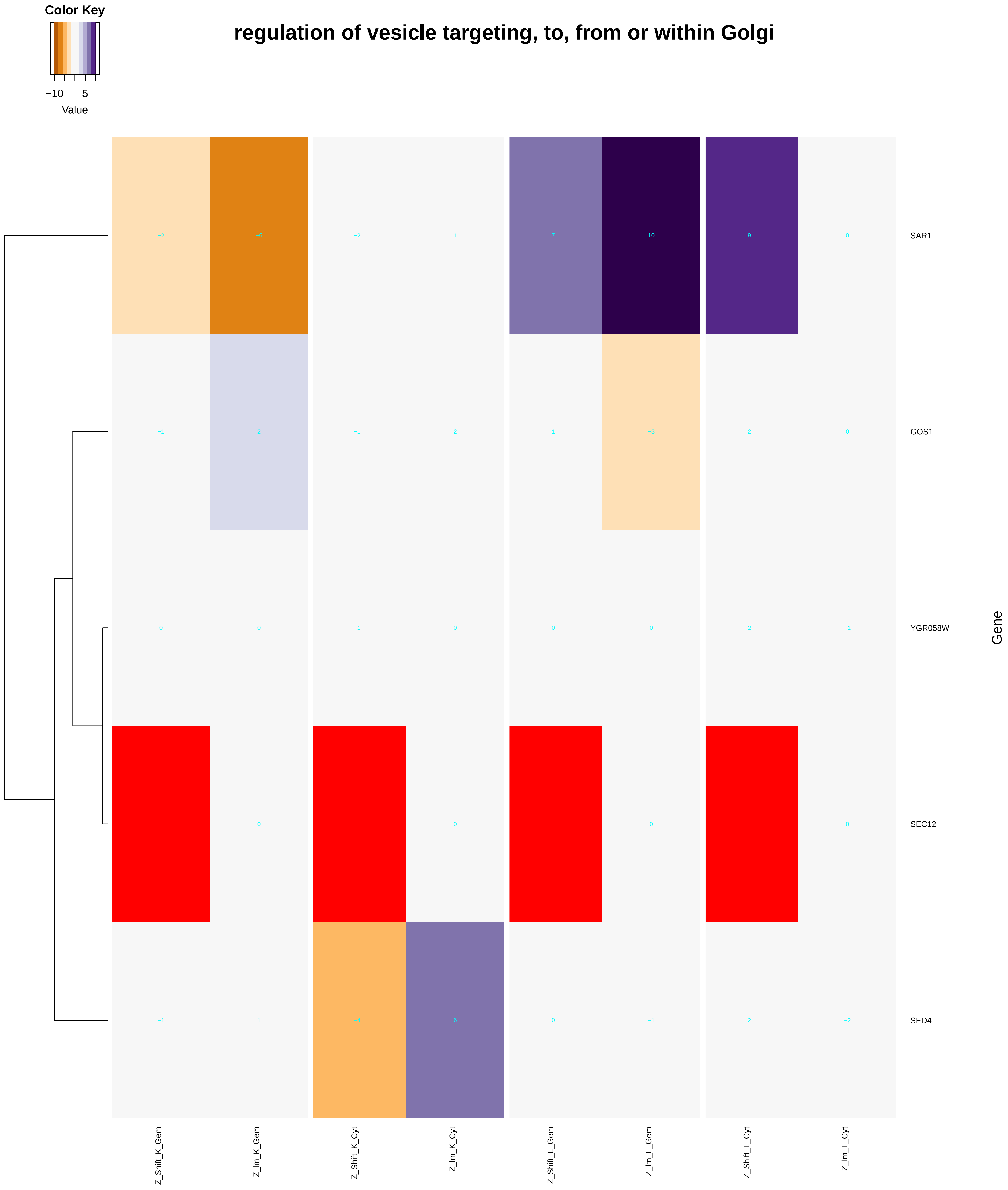
regulation of vesicle targeting, to, from or within Golgi
Color Key
−10
5
Value
SAR1
GOS1
YGR058W
SEC12
SED4
Gene
Z_Shift_K_Gem
Z_Im_K_Gem
Z_Shift_K_Cyt
Z_Im_K_Cyt
Z_Shift_L_Gem
Z_Im_L_Gem
Z_Shift_L_Cyt
Z_Im_L_Cyt

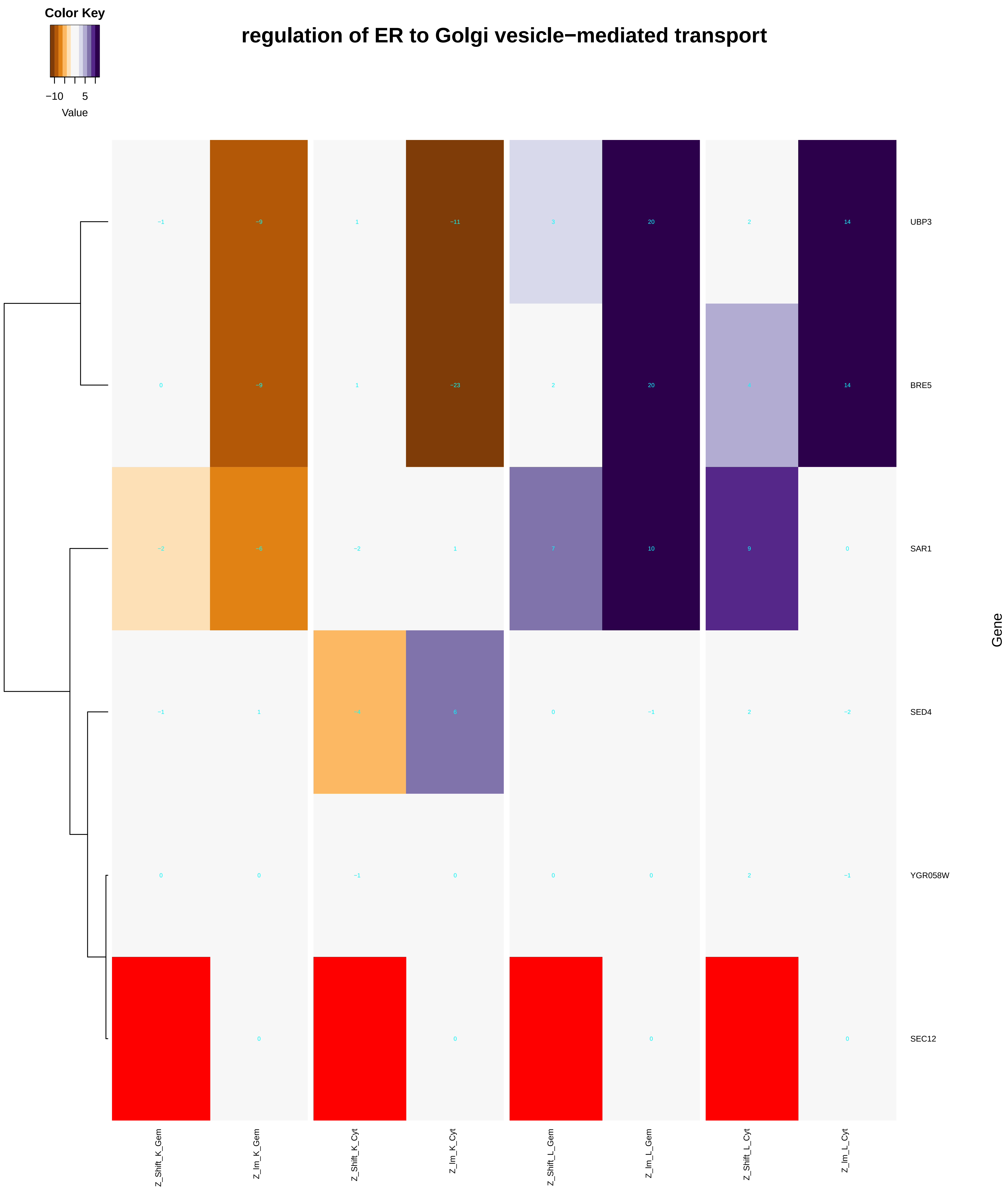
regulation of ER to Golgi vesicle−mediated transport
Color Key
−10 5
Value
UBP3
BRE5
SAR1
SED4
YGR058W
SEC12
Gene
Z_Shift_K_Gem
Z_Im_K_Gem
Z_Shift_K_Cyt
Z_Im_K_Cyt
Z_Shift_L_Gem
Z_Im_L_Gem
Z_Shift_L_Cyt
Z_Im_L_Cyt

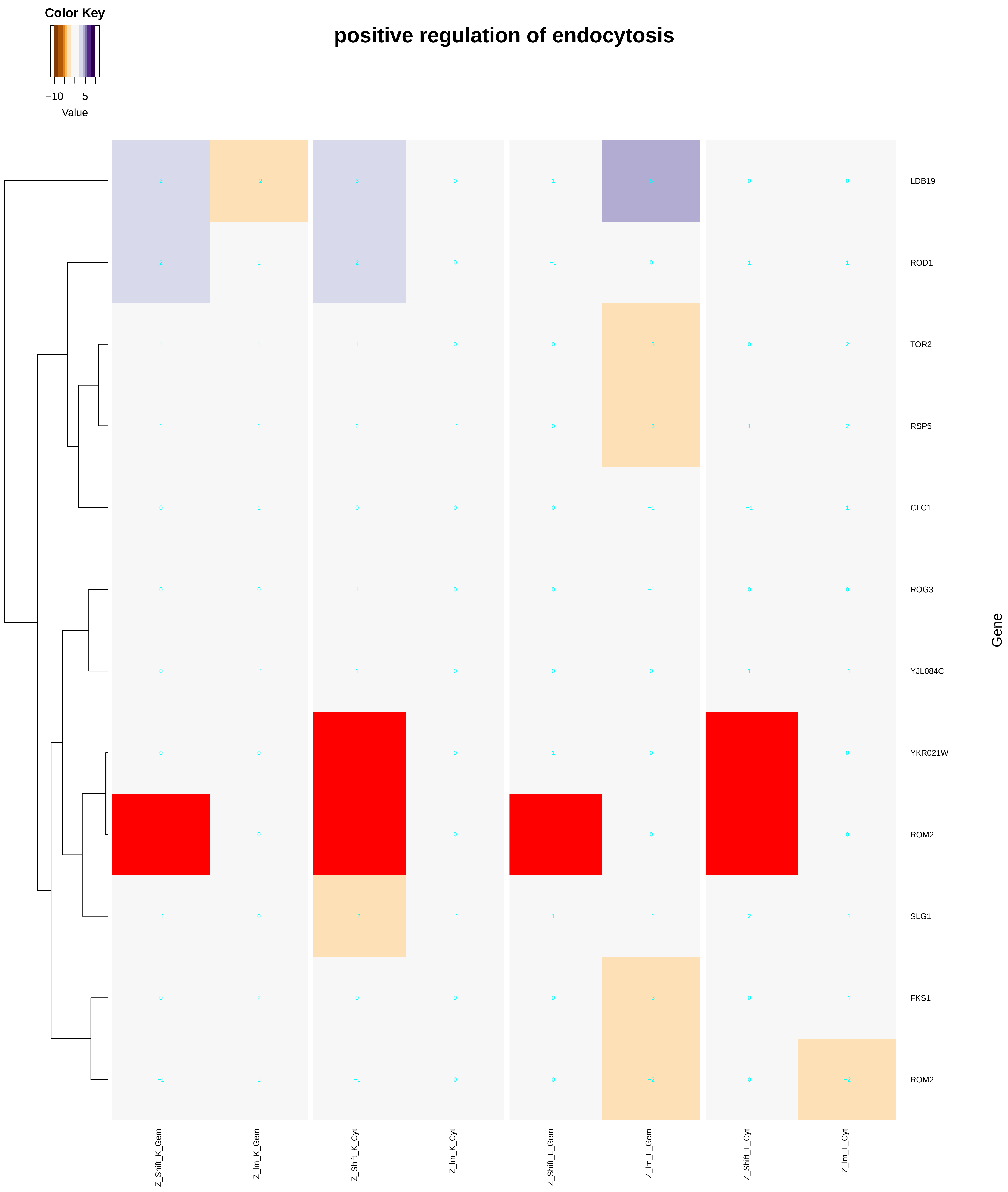
Color Key
−10
5
Value
positive regulation of endocytosis
LDB19
ROD1
TOR2
RSP5
CLC1
ROG3
YJL084C
YKR021W
ROM2
SLG1
FKS1
ROM2
Gene
Z_Shift_K_Gem
Z_Im_K_Gem
Z_Shift_K_Cyt
Z_Im_K_Cyt
Z_Shift_L_Gem
Z_Im_L_Gem
Z_Shift_L_Cyt
Z_Im_L_Cyt

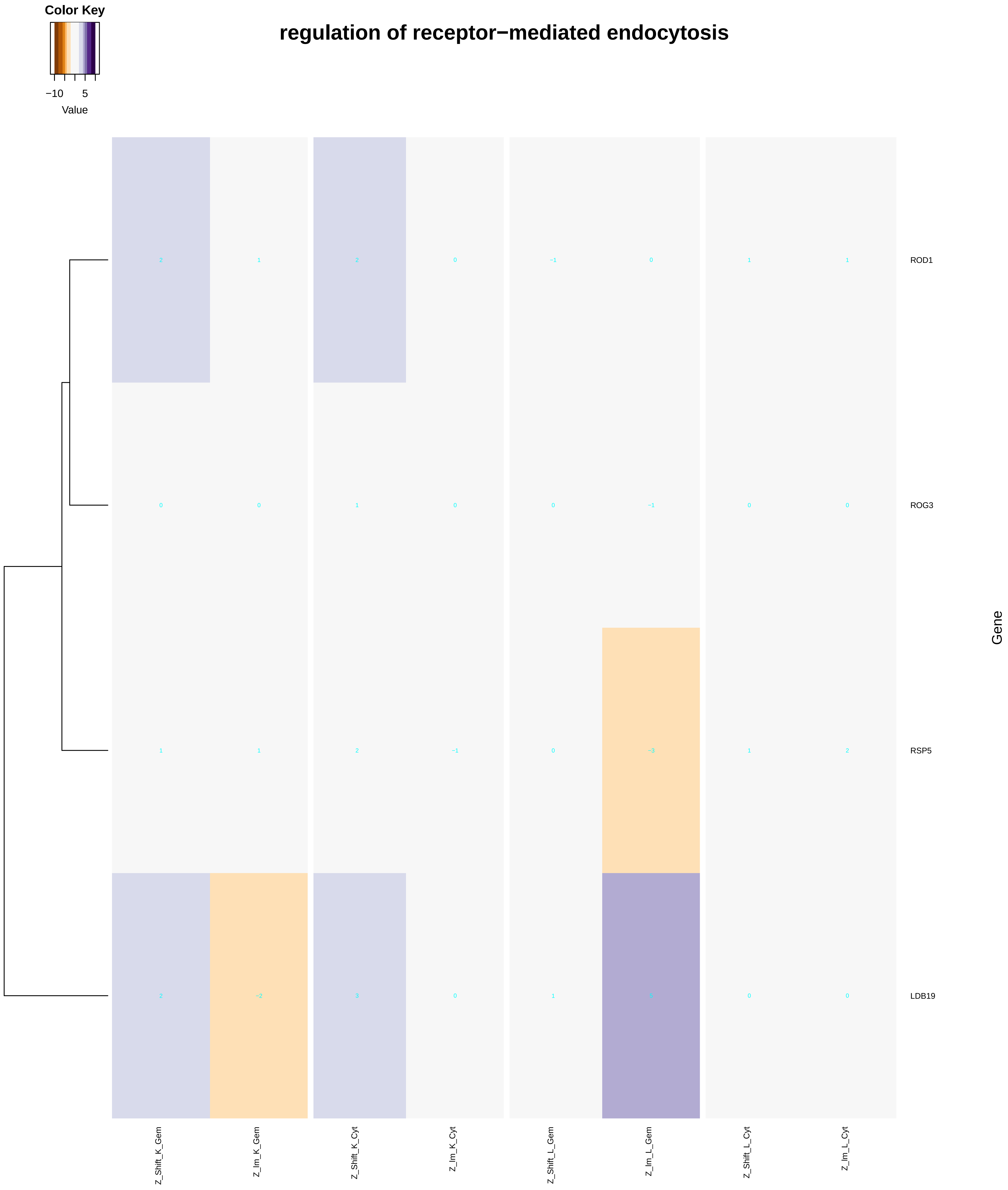
Color Key
−10
5
Value
regulation of receptor−mediated endocytosis
2
1
2
0
−1
0
1
1
ROD1
0
0
1
0
0
−1
0
0
ROG3
1
1
2
−1
0
−3
1
2
RSP5
2
−2
3
0
1
5
0
0
LDB19
Gene
Z_Shift_K_Gem
Z_Im_K_Gem
Z_Shift_K_Cyt
Z_Im_K_Cyt
Z_Shift_L_Gem
Z_Im_L_Gem
Z_Shift_L_Cyt
Z_Im_L_Cyt

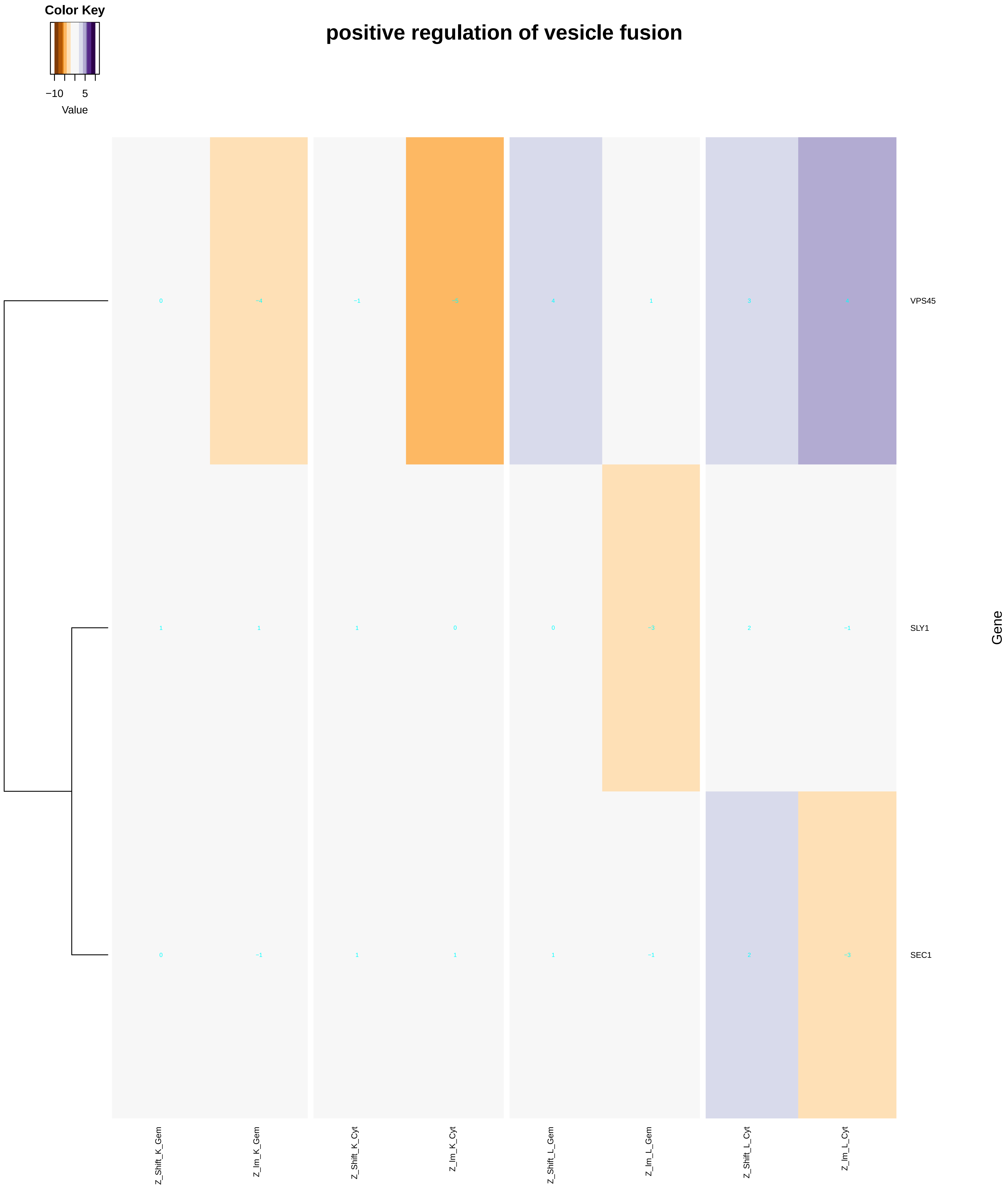
Color Key
−10
5
Value
positive regulation of vesicle fusion
0
−4
−1
−5
4
1
3
VPS45
1
1
1
0
0
−3
2
−1
SLY1
0
−1
1
1
1
−1
2
−3
SEC1
Gene
Z_Shift_K_Gem
Z_Im_K_Gem
Z_Shift_K_Cyt
Z_Im_K_Cyt
Z_Shift_L_Gem
Z_Im_L_Gem
Z_Shift_L_Cyt
Z_Im_L_Cyt

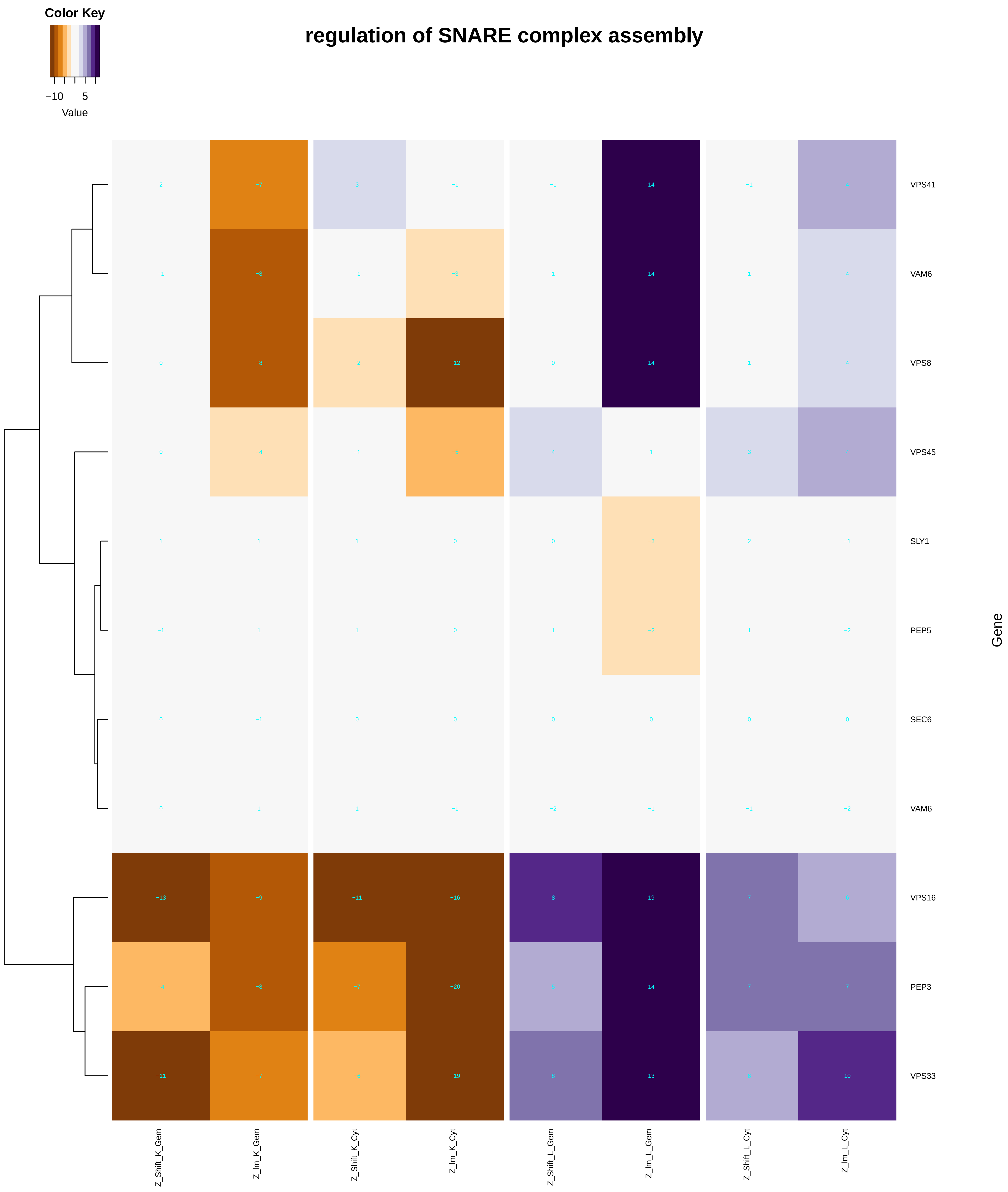

regulation of SNARE complex assembly
Color Key
−10
5
Value
VPS41
VAM6
VPS8
VPS45
SLY1
PEP5
SEC6
VAM6
VPS16
PEP3
VPS33
Gene
Z_Shift_K_Gem
Z_Im_K_Gem
Z_Shift_K_Cyt
Z_Im_K_Cyt
Z_Shift_L_Gem
Z_Im_L_Gem
Z_Shift_L_Cyt
Z_Im_L_Cyt

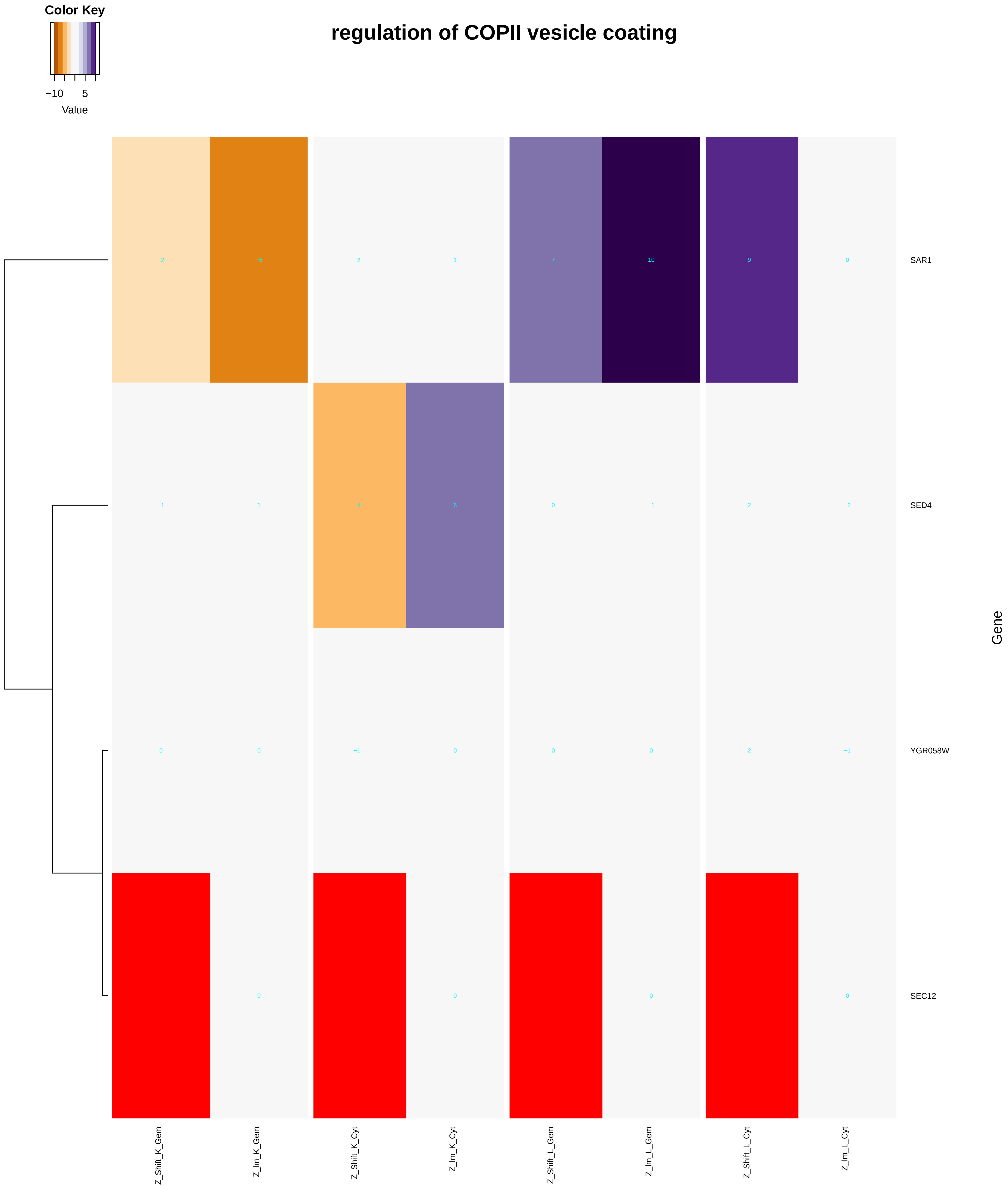
Color Key
−10
5
Value
regulation of COPII vesicle coating
−2
−6
−2
1
7
10
9
0
SAR1
−1
1
−4
6
0
−1
2
−2
SED4
0
0
−1
0
0
0
2
−1
YGR058W
0
0
0
0
SEC12
Gene
Z_Shift_K_Gem
Z_Im_K_Gem
Z_Shift_K_Cyt
Z_Im_K_Cyt
Z_Shift_L_Gem
Z_Im_L_Gem
Z_Shift_L_Cyt
Z_Im_L_Cyt

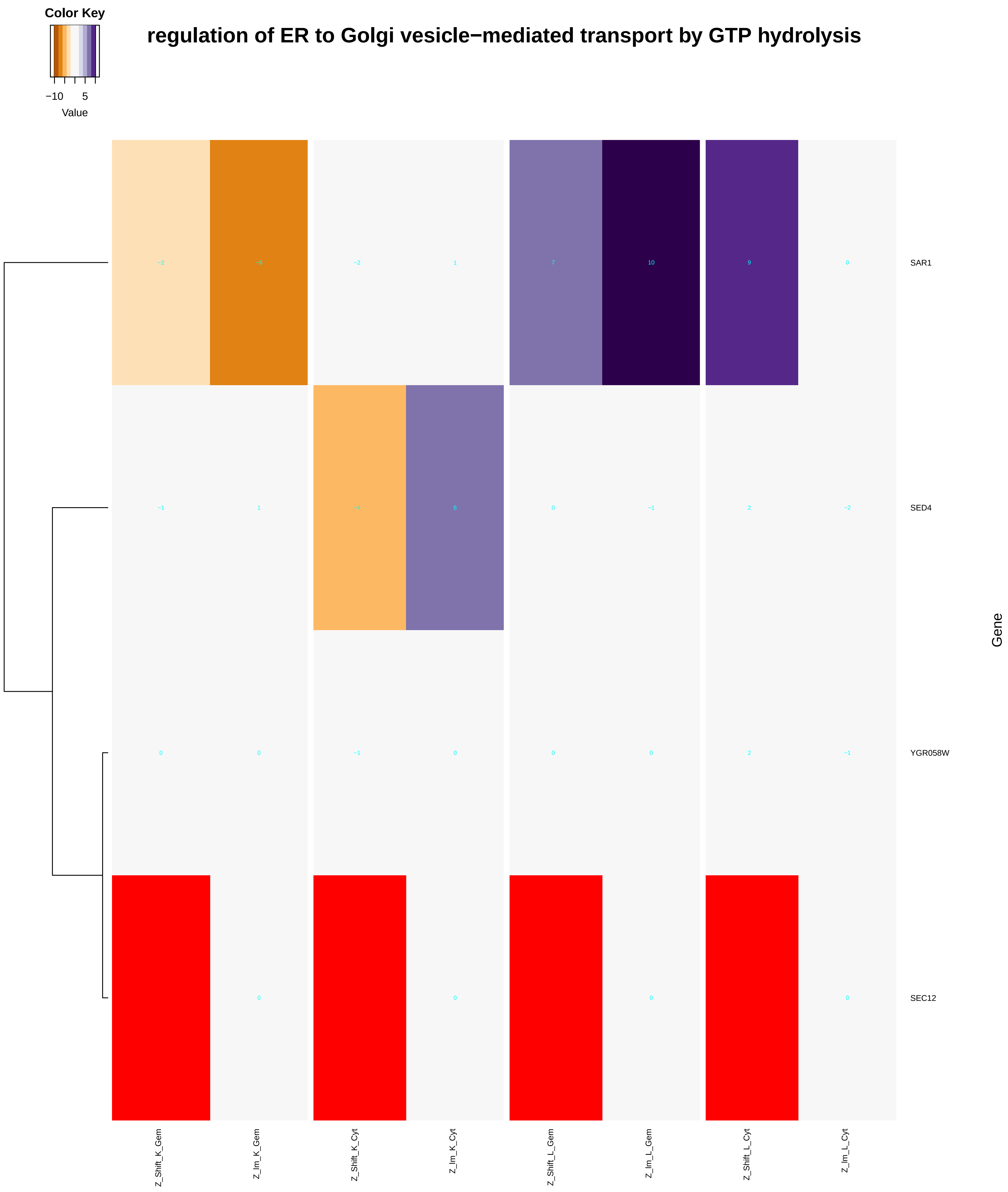

Color Key
−10
5
Value
regulation of ER to Golgi vesicle−mediated transport by GTP hydrolysis
SAR1
SED4
YGR058W
SEC12
Gene
Z_Shift_K_Gem
Z_lm_K_Gem
Z_Shift_K_Cyt
Z_lm_K_Cyt
Z_Shift_L_Gem
Z_lm_L_Gem
Z_Shift_L_Cyt
Z_lm_L_Cyt

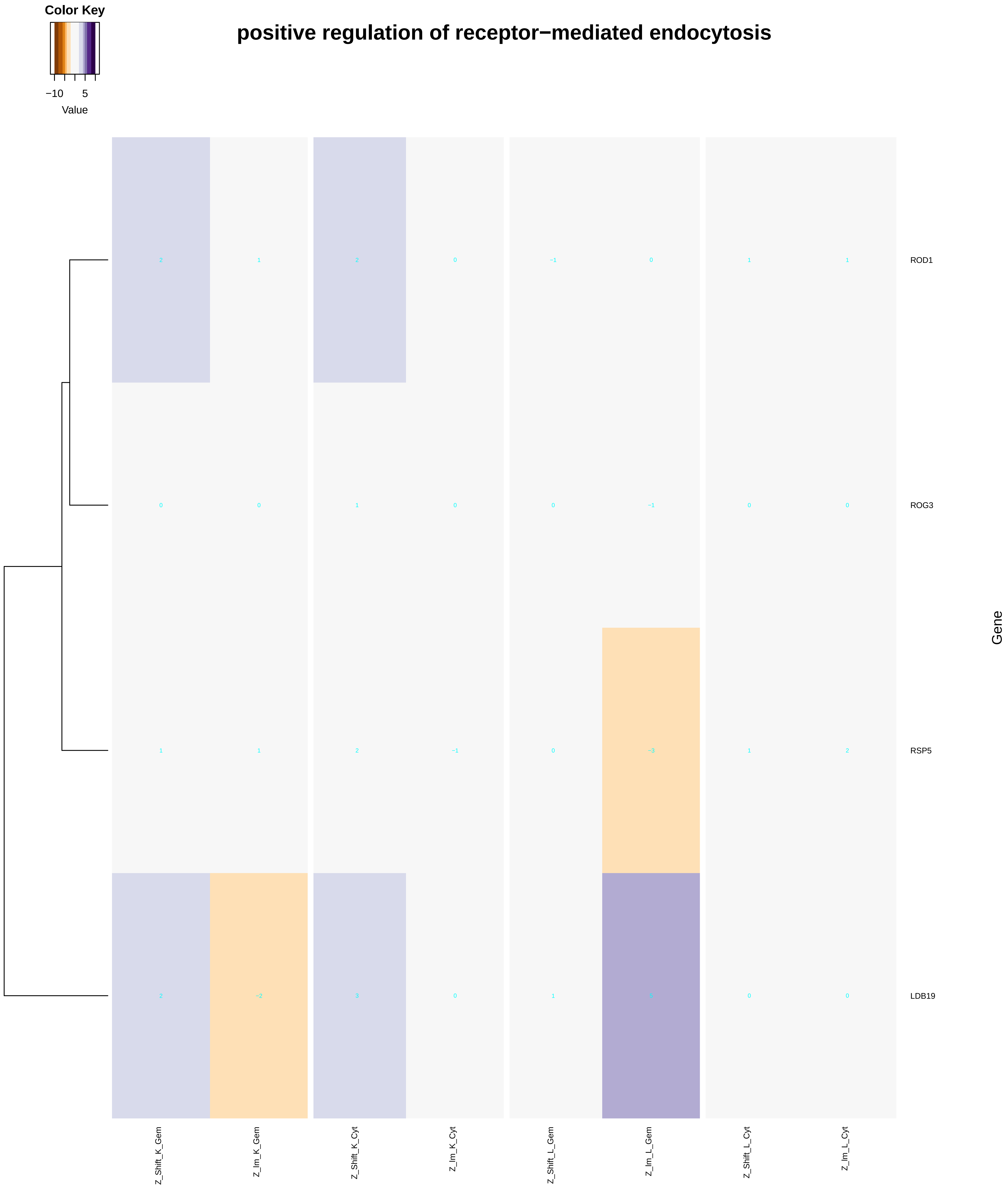

Color Key
−10
5
Value
positive regulation of receptor−mediated endocytosis
2 1 2 0 −1 0 1 1
ROD1
0 0 1 0 0 −1 0 0
ROG3
1 1 2 −1 0 −3 1 2
RSP5
2 −2 3 0 1 5 0 0
LDB19
Gene
Z_Shift_K_Gem
Z_Im_K_Gem
Z_Shift_K_Cyt
Z_Im_K_Cyt
Z_Shift_L_Gem
Z_Im_L_Gem
Z_Shift_L_Cyt
Z_Im_L_Cyt

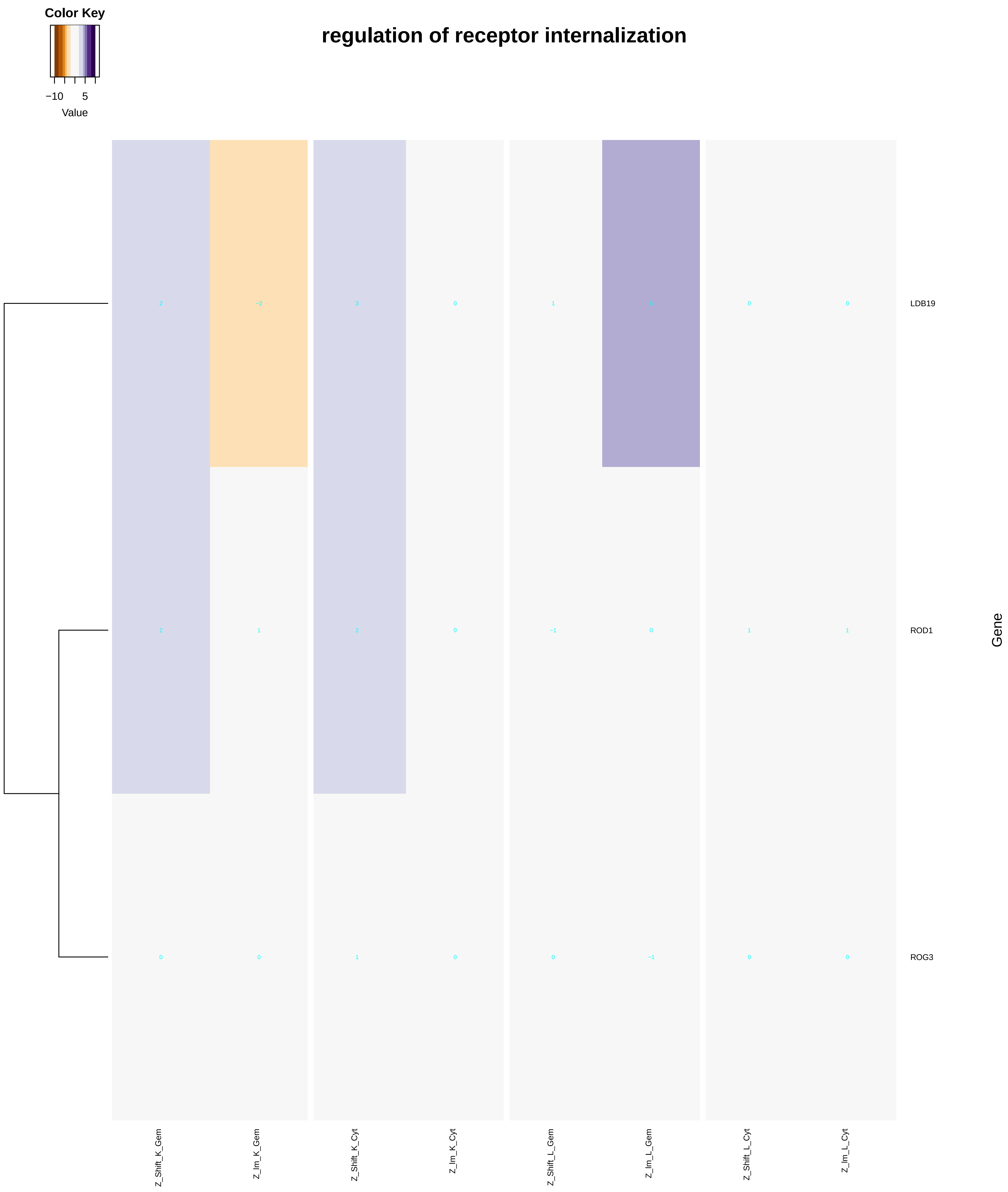

Color Key
−10
5
Value
regulation of receptor internalization
2
−2
3
0
1
5
0
0
LDB19
2
1
2
0
−1
0
1
1
ROD1
0
0
1
0
0
−1
0
0
ROG3
Gene
Z_Shift_K_Gem
Z_Im_K_Gem
Z_Shift_K_Cyt
Z_Im_K_Cyt
Z_Shift_L_Gem
Z_Im_L_Gem
Z_Shift_L_Cyt
Z_Im_L_Cyt

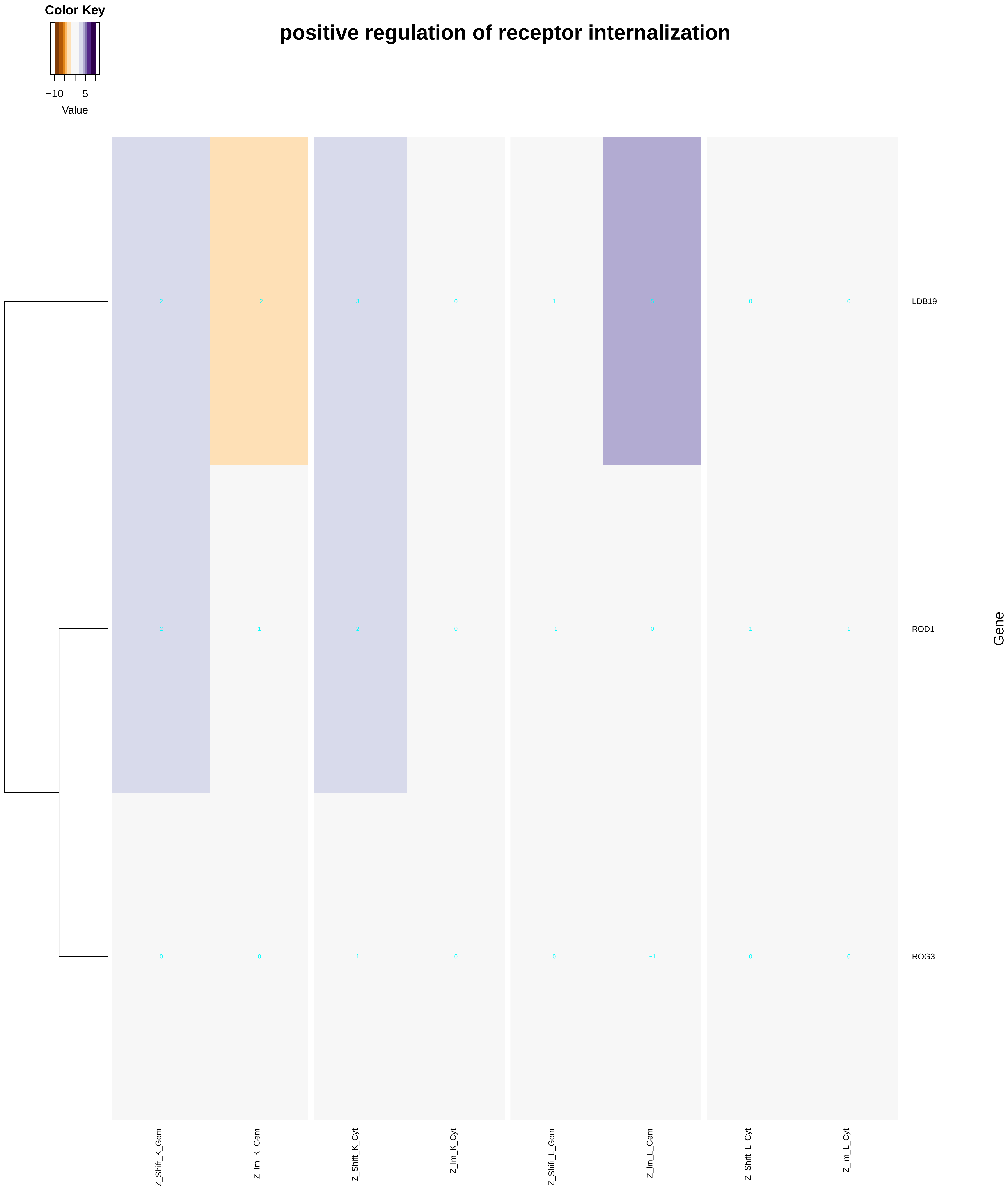
Color Key
−10
5
Value
positive regulation of receptor internalization
2
−2
3
0
1
5
0
0
LDB19
2
1
2
0
−1
0
1
1
ROD1
0
0
1
0
0
−1
0
0
ROG3
Gene
Z_Shift_K_Gem
Z_Im_K_Gem
Z_Shift_K_Cyt
Z_Im_K_Cyt
Z_Shift_L_Gem
Z_Im_L_Gem
Z_Shift_L_Cyt
Z_Im_L_Cyt
